# Supplementary material for: AI-Guided Dual Strategy for Peptide Inhibitor Design Targeting Structural Polymorphs of α-Synuclein Fibrils
Source: Cells. 2025 Dec 3;14(23):1921. doi: 10.3390/cells14231921 (PMC12691048; doi:10.3390/cells14231921)
Supplement: Supplementary file 1 [file cells-14-01921-s001.zip › cells-3973438-supplementary.pdf]

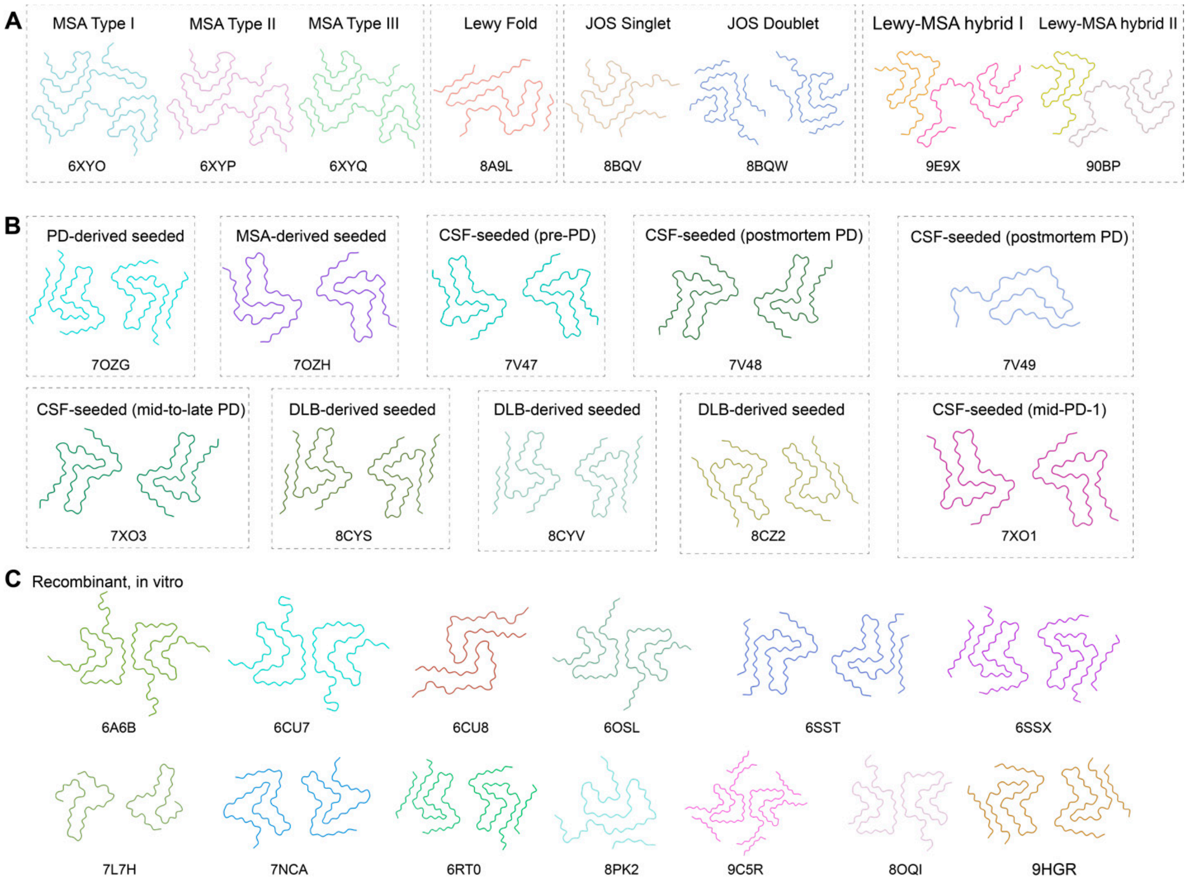

Supplementary Figure S1. Overview of reported α-synuclein fibril polymorphs from disease-derived, seeded, and recombinant assemblies. (**A**) Representative cryo-EM structures of α-syn fibrils from MSA Types I–III, Lewy fold, juvenile-onset synucleinopathy (JOS), and Lewy–MSA hybrid forms (PDB IDs indicated). (**B**) Fibril structures obtained by seeded amplification from CSF or brain-derived material from PD, MSA, DLB, and different disease stages. (**C**) Recombinant α-syn fibrils assembled in vitro under various experimental conditions, illustrating structural variability among laboratory-generated polymorphs.

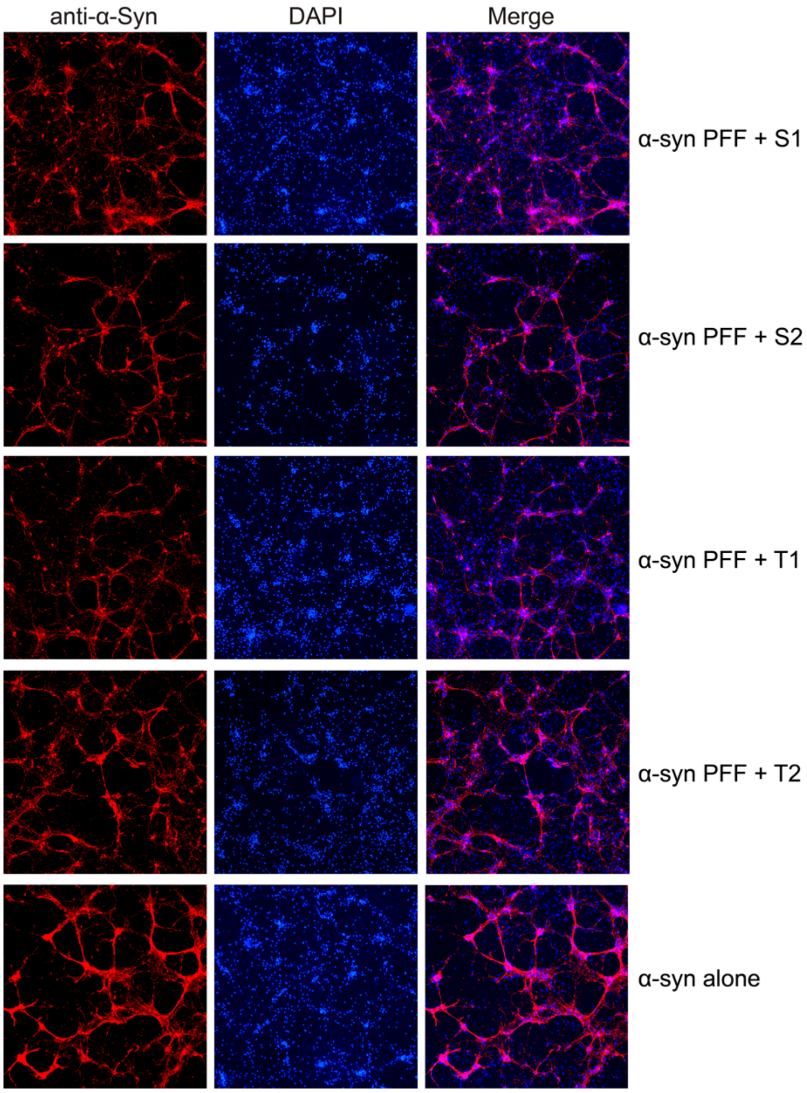

Supplementary Figure S2 Peptide inhibitors suppress *α*-synuclein PFF–induced pathology in primary mouse neurons. Representative immunofluorescence images of primary mouse neurons treated with *α*-syn pre-formed fibrils (PFFs) alone or in combination with peptide inhibitors (S1, S2, T1, T2). Aggregated *α*-syn was detected with an anti–*α*-syn antibody (red), and nuclei were labeled with DAPI (blue). Co-treatment with S1 or T1 markedly reduced *α*-syn accumulation, while S2 and T2 showed moderate inhibition.

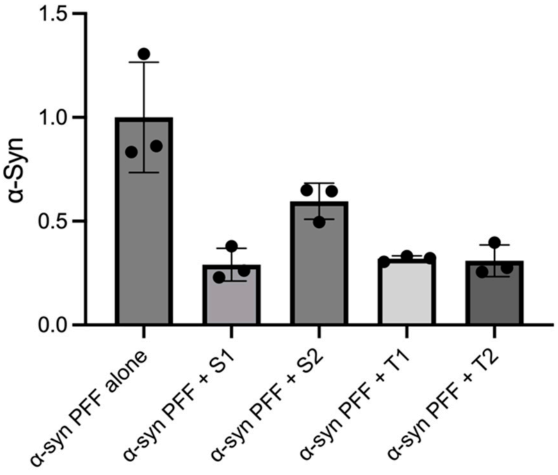

Supplementary Figure S3. Quantification of α-synuclein immunofluorescence intensity in primary neurons treated with peptide inhibitors. Normalized fluorescence intensity of intracellular α-syn aggregates in primary mouse neurons exposed to α-syn PFFs alone or co-treated with peptide inhibitors (S1, S2, T1, T2). Values represent mean ± SEM from three independent cultures. S1 and T1 significantly reduced α-syn accumulation compared with the PFF-only condition, whereas S2 and T2 showed moderate effects (one-way ANOVA with post hoc analysis).

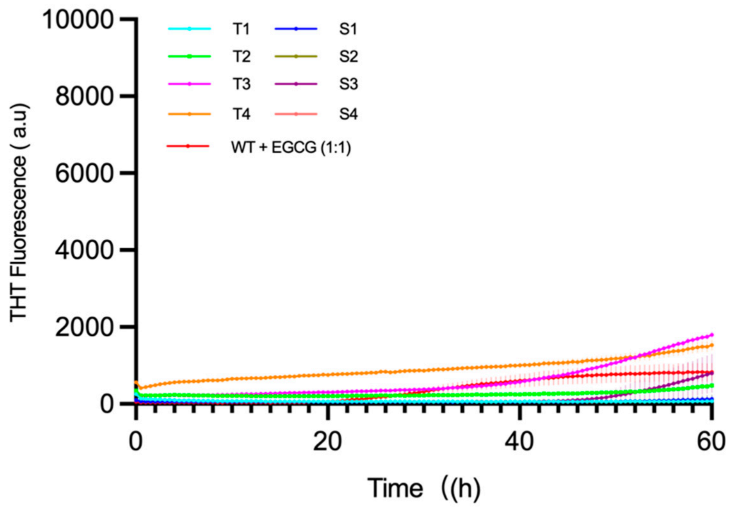

Supplementary Figure S4. ThT fluorescence of peptide-only controls and EGCG inhibition of α-synuclein fibrillation. ThT assays were performed to evaluate intrinsic fluorescence or self-aggregation of the designed peptides in the absence of α-synuclein. All peptides (T1–T4, S1–S4) exhibited baseline signals comparable to buffer controls, indicating no interference with ThT readouts. As a positive control, EGCG markedly reduced ThT fluorescence when co-incubated with α-synuclein, confirming effective inhibition of fibril formation. Data are presented as mean ± SEM from three independent experiments.

Table S1. Peptide Sequences, Total Scores, and Scores Per Residue Generated by ProteinMPNN.

Binder Name	Sequence	Total Score	Score per residue
T1	TALLQR	-5.1	-0.85
T1-1	TALTLQ	-5.7	-0.95
T1-2	TAVTQQ	-6.3	-1.05
T1-3	AALVQL	-7	-1.17
T1-4	TPALQR	-7.5	-1.25
T1-5	AALTQL	-8.2	-1.37
T1-6	DAVTQR	-8.5	-1.42
T1-7	TAATQR	-9.3	-1.55
T1-8	TLVTQL	-10.2	-1.7
T1-9	TAVTSL	-11.5	-1.92
T2	TSLQPLP	-6.8	-0.971
T2-1	TSTVQTP	-7.4	-1.057
T2-2	TEPEETV	-7.9	-1.129
T2-3	SDTVPTK	-8.3	-1.186
T2-4	DDLEPLV	-8.9	-1.271
T2-5	TELQPKK	-9.1	-1.3
T2-6	DSTEQTK	-9.8	-1.4
T2-7	TSTQPTK	-10.2	-1.457
T2-8	TETEELK	-10.8	-1.543
T2-9	DELVPTP	-11.5	-1.643
T3	TAVVQVGA	-7.6	-0.95
T3-1	TAVVQLLG	-8.1	-1.01
T3-2	TSPSLLGR	-8.6	-1.08
T3-3	VSVVVVGG	-8.9	-1.11
T3-4	VAAVLVLG	-9.3	-1.16
T3-5	TVPQQVLA	-9.8	-1.23
T3-6	TAPSQAGR	-10.4	-1.3
T3-7	TAVVQLLA	-11	-1.38
T3-8	TAVQQAGA	-11.4	-1.43
T3-9	TAVSVLGG	-12.2	-1.53
T4	SPASVAAGGFSAR	-13.2	-1.015
T4-1	SPAAIGARVFSLG	-13.8	-1.062
T4-2	SASLVGARRQSAG	-14	-1.077
T4-3	AAVAVGGRVRSAR	-14.5	-1.115
T4-4	SAVSLGAAVRSGR	-14.9	-1.146
T4-5	SPVSVAGGRFSGL	-15	-1.154
T4-6	SASALGGGRRVGL	-15.4	-1.185
T4-7	DVVLVGGGRRVGG	-15.6	-1.2
T4-8	SAASLVGRRQSGG	-16.2	-1.246
T4-9	SPSSVVGRRQSAL	-18	-1.385

Table S2. Peptide Binder Sequences and Pseudo Perplexity from PepMLM.

Binder Name.	Sequence	Pseudo Perplexity
S1	APVVQR	5.564072144
S1-1	APLTQQ	8.922786261
S1-2	TAVVSQ	12.58461485
S1-3	DALLQQ	8.279778046
S1-4	TALLQL	7.25509072
S1-5	TPVLSR	8.162517604
S1-6	TAVTQL	9.930928705
S1-7	TALLQR	11.83062704
S1-8	DALTQR	10.90199888
S1-9	TALLLQ	6.667298666
S2	TELVPLP	8.254263642
S2-1	SSPQPLK	10.00839498
S2-2	DSLEPLP	18.22475198
S2-3	TETVQKV	18.04626184
S2-4	TSTEETV	15.82665221
S2-5	DELVQLK	15.73130404
S2-6	DDLQQKP	18.07948323
S2-7	TSLEPLV	16.65450763
S2-8	DSLVETP	10.2406624
S2-9	TELQPTK	17.17601525
S3	TAAVQAGG	6.011459622
S3-1	TVPVQLTG	8.473778287
S3-2	GAPVQVTR	10.36002073
S3-3	TAVVQAGA	7.145526888
S3-4	TVVQQALA	7.013459623
S3-5	TVVSLVTG	10.20259754
S3-6	TVVQQATR	10.02102737
S3-7	VVVVQLGA	18.57365161
S3-8	TVVQQAGA	9.485377516
S3-9	VVVSQVGA	10.16956025
S4	AVVAVAAARQSGR	8.92050351
S4-1	DASALAARVQSAG	9.776013719
S4-2	SASAIAARRQVAG	9.607311923
S4-3	DVSSLVGGGFVGR	9.578545932
S4-4	SPALVVGGGRSGG	14.3659403
S4-5	DVVAVVAGGQSAR	18.88668571
S4-6	SAALLAAGVQGAR	9.591934752
S4-7	SVAAVGGGVFGGL	10.7474419
S4-8	SAAAIGARGQGAG	12.17773135
S4-9	DPSAVVARVRVLL	10.58438387

*Lower ProteinMPNN design scores and Rosetta ΔG values (approx. −20 to −35 kcal/mol) indicate more favorable interfaces, while lower PepMLM pseudo-perplexity reflects higher sequence plausibility.
